# The biological age of the heart is consistently younger than chronological age

**DOI:** 10.1038/s41598-020-67622-1

**Published:** 2020-07-01

**Authors:** Sofia Pavanello, Manuela Campisi, Assunta Fabozzo, Giorgia Cibin, Vincenzo Tarzia, Giuseppe Toscano, Gino Gerosa

**Affiliations:** 1 Unit of Occupational Medicine, Department of Cardiac, Thoracic, and Vascular Sciences and Public Health, University Hospital of Padova, Via Giustiniani, 2, 35128 Padua, Italy; 2 Lifelab Program, Consorzio Per La Ricerca Sanitaria-CORIS, Veneto Region, Via Giustiniani 2, 35128 Padua, Italy; 3 Cardiac Surgery Unit, Department of Cardiac, Thoracic, and Vascular Sciences and Public Health, University Hospital of Padova, Via Giustiniani, 2, 35128 Padua, Italy

**Keywords:** Biomarkers, Senescence, Epigenetics

## Abstract

Chronological age represents the main factor in donor selection criteria for organ transplantation, however aging is very heterogeneous. Defining the biological aging of individual organs may contribute to supporting this process. In this study we examined the biological age of the heart [right (RA)/left atrium (LA)] and peripheral blood leucocytes in the same subject, and compared these to assess whether blood mirrors cardiac biological aging. Biological aging was studied in 35 donors (0.4–72 years) by exploring mitotic and non-mitotic pathways, using telomere length (TL) and age-dependent methylation changes in certain CpG loci (DNAmAge). Heart non-mitotic DNAmAge was strongly younger than that of both blood (− 10 years, *p* < 0.0001) and chronological age (− 12 years, *p* < 0.0001). Instead, heart and blood mitotic age (TL) were similar, and there was no difference in DNAmAge and TL between RA and LA. DNAmAge negatively correlated with TL in heart and blood (*p* ≤ 0.01). Finally, blood and heart TL (*p* < 0.01) and DNAmAge (*p* < 0.0001) were correlated. Therefore, blood can be a proxy indicator of heart biological age. While future investigation on post-transplant graft performance in relation to biological aging is still needed, our study could contribute to opening up novel basic and clinical research platforms in the field of organ transplantation.

## Introduction

The number of patients suffering from end-stage organ failure that are waiting for a transplant is steadily increasing, but the existing organ shortage does not allow all patients to benefit from this optimal therapeutic option. Current strategies to face organ shortages demand a critical re-examination of donor eligibility, in order to include the elderly^1^.

Although chronological age represents the main factor in donor selection for cardiac transplantation, a great heterogeneity in aging trajectories and health outcomes occurs in people of the same age^2^. Defining the biological age of organ tissue may contribute to supporting this process. People do not age at the same rate, and some of us age much more dramatically than others. Genetic and environmental factors can contribute to biological aging, which means that people may be affected differently, appearing younger or older than their birth date may predict^3–5^. Consequently, age, when measured chronologically, may not be a reliable indicator of the rate of physiological breakdown of the body or organs. Indeed, individual organ systems, cells, organelles, and molecules within individuals may age at significantly different rates^6^. Therefore, it can be postulated that even the heart may have a different aging profile to the body.

At the cellular level, organ aging may be mediated through mitotic and/or non-mitotic pathways^7^. Mitotic—or replicative—cellular aging can be measured using telomere length (TL). Telomeres are non-coding DNA sequences that cap chromosomes and are required for cell division and survival^8^. TL shortens progressively with cell division, indicating a critical threshold of a cell’s proliferative capacity, a process called ‘cellular senescence’^9^. TL measured in peripheral blood leucocytes (LTL) decreases with age and it is considered an indicator of biological age^10^. Shorter LTL controlling for age in turn predicts higher morbidity and mortality rates^11,12^. A powerful emerging marker of non-mitotic cellular aging is epigenetic age, also defined as DNA methylation age (DNAmAge)^13,14^. The advent of epigenome-wide high-throughput sequencing analyses has led to a successful identification of a large number of genomic sites highly associated with age^15^. To support the clinical translational power of these remarkable findings, age-predicting models have been developed and validated for an accurate “biological age” estimation^15–17^. An “epigenetic clock” has been created, with unprecedented accuracy for DNAmAge estimation with an average error of only 3.6 years^15–17^. Such models were based on DNA mainly derived from blood circulating leucocytes as they represent an easily available source^15–18^. Several multiple age-prediction statistical models exist to determine the age of a person that are based on the age-dependent methylation changes in certain CpG loci^15–18^. In this study, we applied the prediction model proposed by Zbieć-Piekarska et al.^17^. Besides our research group^19^ others have also used this method^20,21^. This method was developed on data from five CpG sites, to increase the practicability of these tests, and used the locus-specific technology pyrosequencing which having the potential for multiplexing, makes the technical analysis achievable in few hours and reduce the cost. Additionally, this method shows high correlation between DNAmAge and chronological age equivalent to those from Horvath^15^ and Hannum et al.^16^ which are considered as reference methods. Furthermore, the discrepancy between DNAmAge and chronological age, defined as age acceleration (AgeAcc)^22^, provides information regarding the speed of the epigenetic clock^22^ and is considered a reliable clinical predictor of morbidity and mortality^23^. Environmental factors can also impact on age acceleration and include cumulative lifetime stress^24^, pollution^25^, and dietary and lifestyle factors^26^. In our previous work we observed, after 60 days of intensive relaxing practices, a significant reduction of stress hormones (cortisol, ACTH, copeptin, epinephrine and norepinephrine) together with a decrease in DNAmAge^19^. The understanding of a donor’s organ biological age might be of paramount importance in the prediction of potential outcomes of its transplantation. Therefore, apart from the usual parameters evaluated to verify the quality of a donor’s organs, it may become essential to also establish their degree of biological aging.

This study has two main objectives:

1. To determine the biological age of the heart and of peripheral blood leucocytes, by measuring the mitotic (TL) and the non-mitotic epigenetic age (DNAmAge)
2. To compare blood and heart, in order to assess the reliability of blood as an accurate indicator of heart biological age

## Results

### Biological age of cardiac atrial tissues and blood by DNAmAge and TL

Donors’ Characteristics are summarized in Table 1.

**Table 1:** Donors’ characteristics and myocardial protection techniques.

**N**	35
**Age (years)**	51 (0.4–72)
**Male (n, %)**	27 (77%)
Smokers	12 (34%)
Former smokers	1 (3%)
Non-smokers	22 (63%)
**Comorbidities**	
Arterial hypertension	8 (23%)
Diabetes	2 (6%)
Dyslipidemia	4 (11%)
**Infections**	
Cytomegalovirus	22 (63%)
Toxoplasma	34 (97%)
Epstein barr virus	11 (31%)
**Blood parameters**	
Leucocytes (n * 10^3^/µl)	12.6 (5.78–25.4)
Creatinine (mg/dL)	0.93 (0.27–4.65)
Glucose (mg/dL)	130 (54–252)
**Organ care system (n, %)**	7 (20%)
Cold ischemic time (min)^a^	29 (20–38)
Time in OCS (min)^b^	265 (160–360)
**Cold Cardioplegia (n, %)**	28 (80%)
Cold Ischemic Time (min)^c^	220 (31–330)

Data are expressed in median (range) or number (percentage).

^a^For the OCS, the time of cold ischemia is obtained by calculating the time from aortic cross clamping at the donor site to the aortic de-clamping at the recipient site minus the time the organ was inserted in the OCS device.

^b^While in OCS, the heart is beating and the coronary flow is guaranteed by an extracorporeal blood perfusion.

^c^Cold Ischemic Time: time (min) from aortic cross clamping at the donor site to the time (min) of recipient aorta declamping.

**Figure 1:**
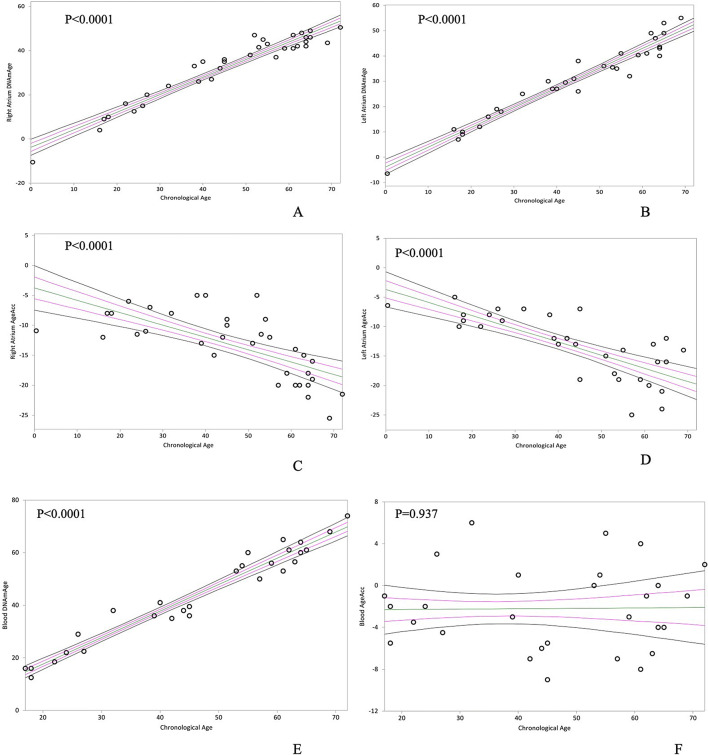
In (**A**) and (**B)**, non-parametric linear regression plots showing correlation between donor chronological age and DNAmAge of the right atrium (RA) and the left atrium (LA) (Kendall’s rank correlation coefficient tau b for RA = 0.741, for LA = 0.852). In (**C)** and (**D)**, non-parametric linear regression plots showing the correlation between AgeAcc and chronological age of RA in A and LA donors in B (Kendall’s rank correlation coefficient tau b = − 0.589 and tau b = − 0.578). In (**E)** and (**F)**, non-parametric linear regression plots showing the correlation between DNAmAge of the circulating blood leucocytes (indicated as “blood age”) and the chronological age of the donor in A (Kendall’s rank correlation coefficient tau b = 0.842), whereas in B no correlation is shown between chronological age and blood AgeAcc (Kendall’s rank correlation coefficient tau b = 0.011).

In Tables 2 and 3 the epigenetic non-mitotic DNAmAge and the mitotic TL age of the right (R–) and left (L-) atrial tissue (–A) and blood are shown. RA and LA DNAmAge, and its difference with chronological age, designated as AgeAcc, were remarkably lower than chronological age (Table 2, median RA and LA AgeAcc = − 12 and − 13 years). This measure was even lower than that of blood (Table 2, median AgeAcc RA versus Blood and LA versus Blood = − 12 vs − 3 and − 13 vs − 3 years, *p* < 0.0001). Table 2 also shows that RA and LA have equivalent DNAmAge values (Table 2, median AgeAcc − 12 vs − 13 years, *p* = 0.948). Furthermore, RA and LA AgeAcc decreased with chronological age (Fig. 1C,D, *p* < 0.0001 and *p* < 0.0001), while blood AgeAcc did not correlate with chronological age (Fig. 1F, *p* = 0.937). As expected, blood DNAmAge (Fig. 1E) was confirmed to be highly related to donor chronological age (*p* < 0.0001). Similarly, RA (*p* < 0.0001) and LA (*p* < 0.0001) DNAmAge were highly associated with chronological age (Fig. 1A,B).

**Table 2:** DNAmAge and AgeAcc of donors’ RA, LA and blood.

Donors	DNAmAge (years)			AgeAcc (years)		
	RA	LA	Blood	RA	LA	Blood
	N = 34	N = 32	N = 28	N = 34	N = 32	N = 28
Median	38^a^	32^a^	46	− 12^b^	− 13^b^	− 3
Min/max	− 11/51	− 7/55	13/72	− 26/− 5	− 25/− 5	− 9/6

*RA* right atrium, *LA* left atrium.

^a^Mann–Whitney U test: Right Atrium DNAmAge versus Blood DNAmAge (Two sided *p* = 0.0172) and Left Atrium DNAmAge versus Blood DNAmAge (Two sided *p* = 0.0034).

^b^Mann–Whitney U test: right atrium versus blood (AgeAcc) and left atrium versus blood (AgeAcc); Two sided *p* < 0.0001.

**Table 3:** Telomere length of donors’ RA, LA and blood.

Donors	Telomere length (T/S)		
	RA	LA	Blood
	N = 34	N = 32	N = 23
Median	1.35	1.41	1.41
Min/max	0.88/2.11	0.58/2.46	0.91/2.46

*RA* right atrium, *LA* left atrium.

Finally, epigenetic DNAmAge and TL were not influenced by the modality of myocardial protection (cold cardioplegia or OCS) after organ harvesting, nor by the length of cold ischemia (*p* = 0.340 and *p* = 0.450).

**Figure 2:**
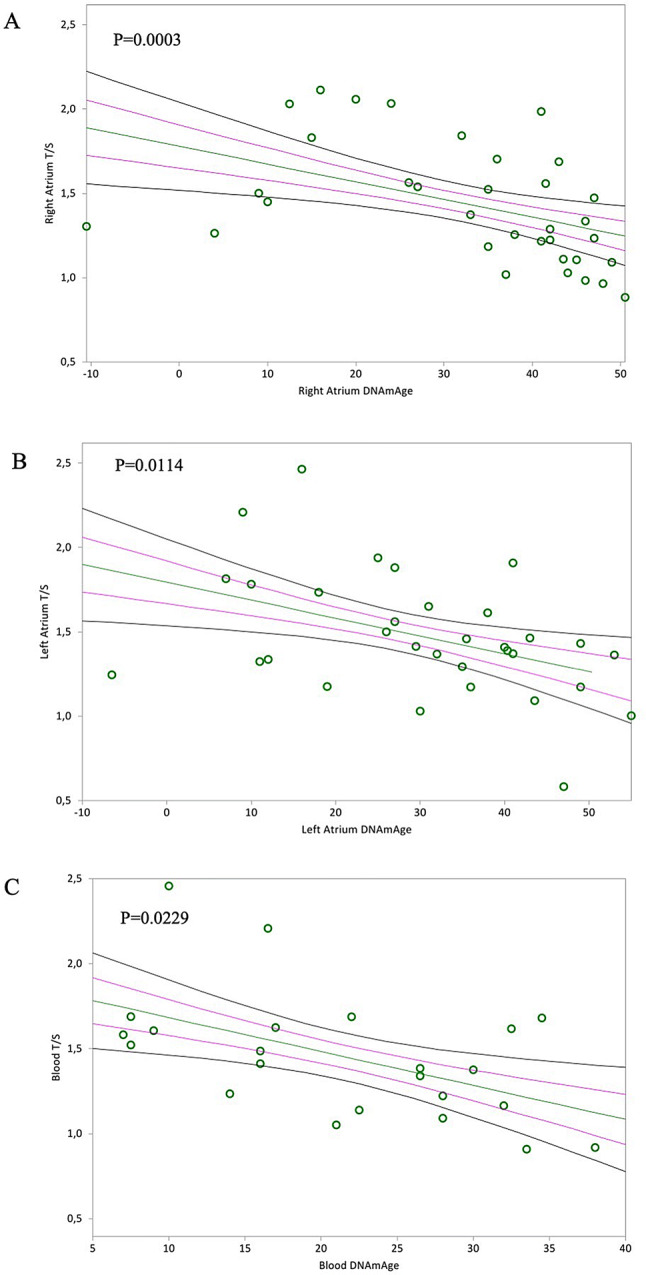
In (**A**) and (**B**), non-parametric linear regression plots showing correlation between donor telomere length (T/S) and donor DNAmAge of the right atrium (RA) and the left atrium (LA) (Kendall’s rank correlation coefficient tau b for RA = − 0.440, for LA = − 0.317). In (**C**), non-parametric linear regression plots showing the correlation between donor telomere length and DNAmAge of the circulating blood leucocytes (indicated as “Blood T/S” and “Blood DNAmAge”) (Kendall’s rank correlation coefficient tau b = − 0.347). Mean, Standard Error (SE) and 95% coefficient intervals (CI) are represented as green, pink and black lines, respectively.

**Figure 3:**
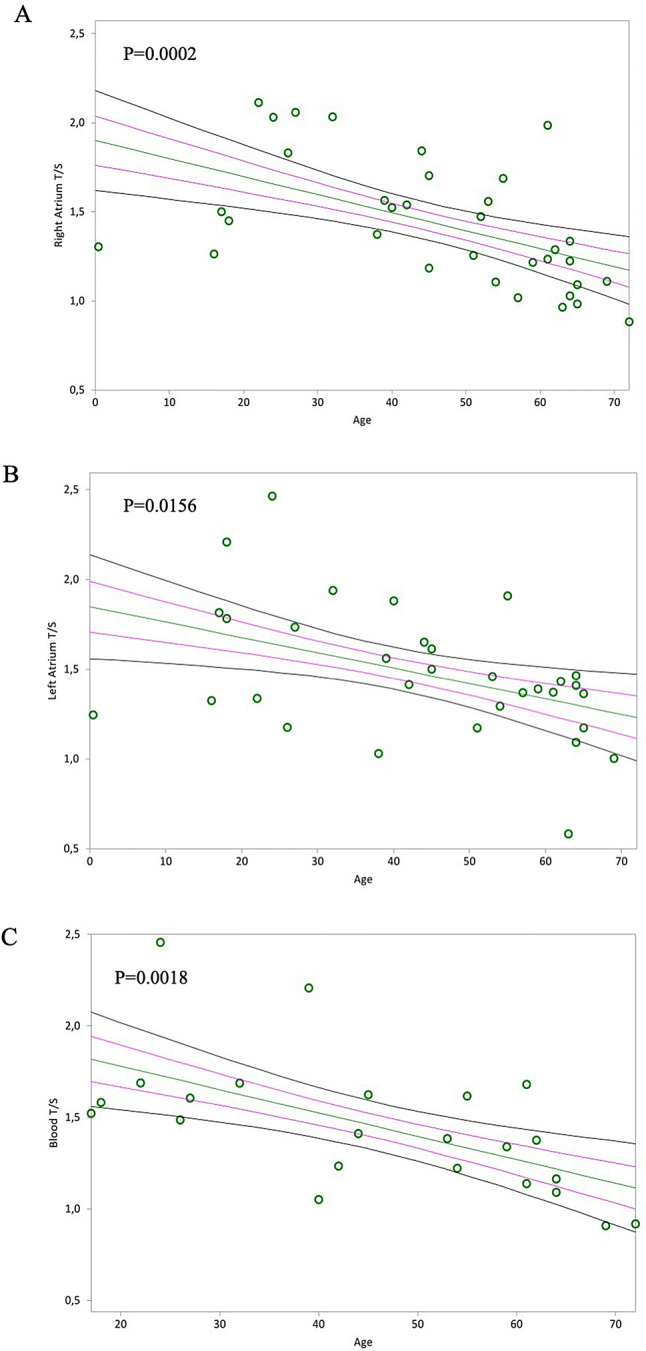
In (**A**) and (**B**), non-parametric linear regression plots showing correlation between telomere length (T/S) of the Right Atrium (RA) and the Left Atrium (LA) and donor chronological age (Kendall’s rank correlation coefficient tau b for RA = − 0.446, for LA = − 0.304). In (**C**), non-parametric linear regression plots showing the correlation between telomere length (T/S) of the circulating blood leucocytes (indicated as “blood T/S”) and the chronological age of the donors (Kendall’s rank correlation coefficient tau b = − 0.472). Mean, Standard Error (SE) and 95% coefficient intervals (CI) are represented as green, pink and black lines, respectively.

The mitotic (TL) age of RA, LA, and blood negatively associated with epigenetic non-mitotic DNAmAge (Fig. 2A, *p* = 0.0003; Fig. 2B, *p* = 0.0114 and Fig. 2C, *p* = 0.0229, respectively). Furthermore, as expected, TL of RA, LA and blood leucocytes negatively correlated with chronological age (Fig. 3A, *p* = 0.0002; Fig. 3B, *p* = 0.0156; Fig. 3C, *p* = 0.0018, respectively). However, there was no difference in TL between RA and LA, between RA and blood, nor between LA and blood (Table 3).

In our analysis, we also excluded any effect of leucocyte count on blood DNAmAge and TL measures (data not shown *p* = 0.991 and *p* = 0.312). Gender also did not influence DNAmAge, AgeAcc and TL of RA (data not shown *p* = 0.729; *p* = 0.984; *p* = 0.887), LA (*p* = 0.767; *p* = 0.877; *p* = 0.171) and blood leucocytes (*p* = 0.416; *p* = 0.250; *p* = 0.968).

### Relationship between blood and heart biological age

**Figure 4:**
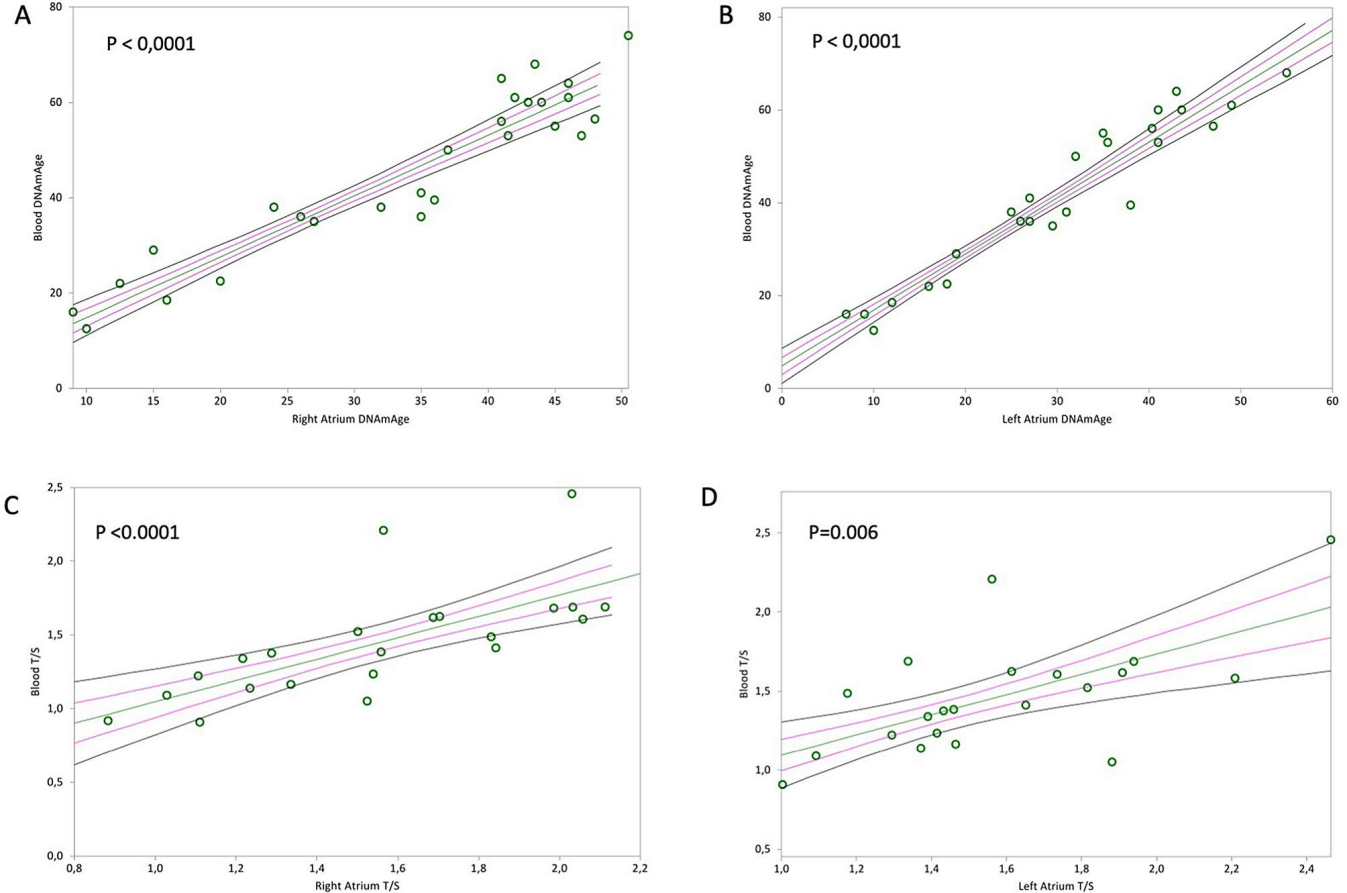
In (**A**) and (**B**), non-parametric linear regression plots showing correlation between donor DNAmAge of the circulating blood leucocytes (indicated as “Blood DNAmAge”) and the DNAmAge of the Right Atrium (RA) and Left Atrium (LA) (Kendall’s rank correlation coefficient tau b for RA = 0.735, for LA = 0.852). In (**C**) and (**D**), non-parametric linear regression plots showing correlation between donor TL of the circulating blood leucocytes (indicated as “Blood T/S”) and the TL of the Right Atrium (RA) and Left Atrium (LA) (Kendall’s rank correlation coefficient tau b for RA = 0.645 and for LA = 0.438). Mean, Standard Error (SE) and 95% coefficient intervals (CI) are represented as green, pink and black lines, respectively.

Blood leucocytes and heart DNAmAge and TL (Fig. 4A,B, Kendall’s rank correlation coefficient tau b for RA = 0.735; *p* < 0.0001 and LA = 0.852; *p* < 0.0001; Fig. 4C,D, Kendall’s rank correlation coefficient tau b for RA = 0.645; *p* < 0.0001 and LA = 0.438; *p* = 0.006) were significantly correlated.

## Discussion

In this study, we have determined the biological age of the heart, specifically of the RA and LA, and of peripheral blood leucocytes, by measuring the mitotic (TL) and the non-mitotic epigenetic age (DNAmAge). We found that DNAmAge, of both atrial tissues (RA and LA), was younger in respect to the chronological age (− 12 years). Furthermore, no significant difference existed between RA and LA, suggesting that, although anatomically diverse and exposed to different physiological conditions, different areas of the heart had the same epigenetic non-mitotic age. Furthermore, the epigenetic age of both RA and LA, was even younger than that of the blood (− 10 years).

DNA methylation is currently the most promising molecular marker for monitoring biological aging and predicting life expectancy^27^. In humans, DNA methylation changes start early in life, as demonstrated by longitudinal studies of infants’ blood^28,29^. Notably, these early epigenetic profiles continue to accumulate changes with the advancement of age, even more so in twins that do not share the same habits and/or environments^30,31^, indicating that aging-associated DNA methylation changes are caused by environmental factors too. In the present study, we demonstrated that biological age of the heart did not reflect the donor’s chronological age, while blood tracked these modifications. This would suggest that while blood is more susceptible to epigenetic changes induced by the interaction of advancing age and environmental factors, the heart is affected by these factors to a lower extent. Furthermore, our results are comparable to those reported by Horvath^15^ where tissues from cardiac ventricular biopsies from 25 patients affected by dilated cardiomyopathy were analyzed, although this was done without comparison with blood from the same subject. It could be also postulated that the presence of stem cells in the cardiac muscle may explain why human heart tissue tends to have a lower DNAmAge. In fact, stem cells are found in relatively large numbers within myocardial tissue and show a DNAmAge close to zero. This can be confirmed by analysis performed on iPS, which is a type of Pluripotent Stem cell artificially derived from a non-pluripotent cell, and which are also significantly younger than the corresponding primary cells^15,32^. However, further investigation is required to elucidate the role of cardiac stem cells in determining epigenetic age of cardiac tissue and to fully understand its discrepancy with chronological age (i.e. the AgeAcc) of the donor. Carefully designed studies will be needed to dig deeper into these issues.

Furthermore, we found that AgeAcc of the heart significantly decreased with increased chronological age, indicating that the epigenetic clock in older hearts reduces its speed of aging. Our results agree with the hypothesis that the ticking rate of the epigenetic clock slows down in later life, as proposed by Horvath^15^. In addition, DNA methylation predicts biological age more efficiently than chronological age. In fact, the rates of epigenetic AgeAcc, have been associated with symptoms of aging, such as frailty and menopause^33,34^, as well as with several aging-associated pathologies including cancer and neurodegenerative diseases^15,35,36^. Moreover, AgeAcc can be used to predict life expectancy because it was shown to predict all-cause mortality independently of common risk factors^22,23^. In other words, our results would suggest that AgeAcc might be the epigenetic clock that mirrors the real biological state of the heart. Prior to the current study however, the implications of biological age determination in the field of organ transplantation has never been explored.

In addition, we found a significant robust negative association between the rise in DNAmAge and the decline in TL of RA and LA, as well as of blood. DNAmAge, therefore, appears to be a promising biomarker in the analysis of different phenotypes of aging. This means that aging research should not focus only on pathways associated with mitotic age, which is classically measured with TL. However, the number of studies investigating the association between TL and epigenetic age is currently very limited. Our findings agree with two previous studies^18,33^ that verified this correlation in blood, while other studies have not found any association^37,38^. The reason for such discrepancy can be related to technical bias and confounding factors in TL measurement, including different methods of DNA extraction and pre-analytical conditions^39,40^. On the other hand, DNA methylation assays, in particular using pyrosequencing, are considered highly robust for biomarker development and for clinical applications^41^. Therefore, DNAmAge can be considered the more promising molecular estimator of biological age than TL^42^.

TL values were comparable between RA, LA and blood leucocytes, suggesting that the mitotic age of the heart is similar to that of blood. Telomeres respond to distinct mechanisms with respect to DNAmAge; TL is a measure of ‘mitotic age’, which is directly modified by cellular division and is induced by factors that control cellular proliferation rates, including inflammation and cell-turnover rates^43,44^. The reason that no difference was detected between cardiac tissue and blood in terms of mitotic (TL) age, is because no particular proliferative stimulus was occurring in the donor hearts, strengthening the concept that the subjects were healthy donors.

Furthermore, with the aim of promptly translating our observations into the clinic, we evaluated the degree of similarity between biological ages of cardiac tissues and blood leucocytes in the same subject. According to our analysis, blood and heart TL and DNAmAge highly correlated, therefore blood can be a proxy indicator of heart biological age. To the best of our knowledge, such correlation for DNAmAge has not been investigated before now. The reason for determining the biological age of blood and trying to find a correlation with that of the heart is clearly explained by the need to identify a simple and reliable test when screening potential donors. In a real clinical scenario, at the donor hospital site, blood samples may be easily acquired and sent for biological age analysis. However, some caution is mandatory since the difference between DNAmAge in blood and heart is at least 10 years. Further studies are therefore needed to optimize the use of blood as a surrogate indicator of heart biological age in clinical practice.

The main strength of our study is that we evaluated non-mitotic epigenetic and mitotic age in tissues obtained from healthy subjects (heart donors) and the results were significant. A limitation of our study could be the small number of subjects enrolled and number of samples collected, which was due to the limited available sources (i.e., donors), represented by donation after brain death (DBD). In addition, in our study, we used atrial tissues of healthy donors with the purpose of demonstrating that cardiac biological age might not reflect the donors’ chronological age. In the current era, a shortage of organs does not allow all patients suffering from end-stage organ failure to have access to a transplant, which remains their optimal therapeutic option. Accepting donors older than 50 y/o has already contributed to widening the pool of available organs, but, to date, there is no evidence that the chronological age of the donors corresponds to the biological status of their organs. Our findings could therefore represent a milestone in the process of donor organ procurement, and thus unquestionably demands a critical review of the currently accepted clinical criteria.

## Conclusion

According to our data, the biological age of cardiac tissues was consistently younger than chronological age, suggesting that the chronological age limit for donors could be extended as it does not reflect the real biological age/status of the heart. Nevertheless, further investigation is needed on post-transplant graft performance and durability in relation to biological age, to investigate long-term effects. This work could contribute to opening a novel basic and clinical research platform in the field of all solid organ transplantation.

## Methods

### Donor heart harvesting, donor blood sampling and transportation to recipient’s site

Over a time span of 17 months (from February 2018 to December 2019), for the purpose of this study, cardiac tissue and blood samples were obtained from 35 heart transplants performed at our Cardiac Surgery Unit (Department of Cardiac, Thoracic, and Vascular Sciences and Public Health, University Hospital of Padua). Median age of enrolled donors was 51 years (from 0.4 to 72 years) and 27 of them were male (77%), all were donors after brain death (DBD). Inclusion and exclusion criteria for donor selection followed current guidelines^1^. At the donor site, hearts were explanted following standard surgical practice. After an initial dose of cardioplegic arrest, myocardial protection and safe organ transportation to the recipient site were obtained either (1) using hypothermia and cold cardioplegia to maintain low levels of cellular metabolism (n = 28 pts) or (2) guaranteeing adequate coronary blood flow in a beating and normothermic heart, using machine perfusion devices (n = 7), such as OCS (Organ Care System, Transmedics Inc.)^45^. For the purpose of this study, a blood sample (3–4 ml) from the donors, was collected in PAXgene tubes (BD Biosciences, USA). Donors’ main characteristics are summarized in Table 1. Our Local Ethical Committee, which is named the Ethical Committee for Clinical Trials of the Province of Padova, approved the study (protocol number 2246P) in accordance with principles of the Helsinki Declaration, allowing a waiver from consent. All methods were carried out in accordance with relevant guidelines and regulations.

### Tissue sampling and storage

Once reached the recipient site, donor’s cardiac tissues were collected. Surgery was performed using a “bicaval technique” for all patients as standard. During surgery, donor atrial walls were trimmed and adapted to recipient atrial cuff for anastomosis, as usual. Two samples of exceeding tissue from right (RA)—by means of venous caval tissue—and left atrium (LA), at least approximately 3 mm^3^, were collected and placed in all protect tissue reagent-RNA Later (Qiagen, Milano, Italy) for DNA/RNA stabilization. All collected samples were then, transferred to our laboratory of Genomic and Environmental Mutagenesis (Department of Cardiac, Thoracic, and Vascular Sciences and Public Health, University Hospital of Padua) for genetic and epigenetic analyses and stored at − 20 °C, until analyses were performed.

### DNAmAge analysis

After DNA extraction from both whole blood and cardiac atrial tissue samples, DNAmAge was determined by analysis the methylation levels from selected markers using bisulfite conversion and Pyrosequencing methodology. This method is based on determination of methylation level of a set of five markers (ELOVL2, C1orf132, KLF14, TRIM59 and FHL2) in genomic DNA^17^, with some modifications due to the fact that the method was completely automated using the PyroMark Q48 Autoprep (Qiagen, Milano, Italy), as we previously described^19^. Details of DNA extraction and DNAmAge analyses are reported in the Supplementary information.

### AgeAcc

AgeAcc was calculated as the difference between the detected DNAmAge of cardiac tissue and blood leucocytes and the chronological age of the donors. The predicted DNAmAge^ was also calculated by regressing DNAmAge on chronologic age and AgeAcc^ by calculating the difference between the predicted DNAmAge^ and chronological age. No difference was detected with results obtained with data from AgeAcc and AgeAcc^, therefore we used both DNAmAge and AgeAcc data.

### Telomere length (TL) analysis

TL was measured after DNA extraction from whole blood and cardiac atrial tissue samples, by quantitative Real-Time PCR as previously described^4^. This assay measures relative TL in genomic DNA by determining the ratio of telomere repeat copy number (T) to single nuclear copy gene (S), i.e. the T/S ratio. The single-copy gene used was human (beta) globin (hbg). Two different pool of DNA were made for TL analyses on DNA extracted from blood and heart tissue samples. A fresh seven points standard curve from the pool, ranging from 40 to 0.625 ng/μl (serial dilutions 1:2), was included in every “T” and “S” PCR run, against a negative control (water). In brief, Qiagility (Qiagen, Milano, Italy) that enables a high-precision PCR setup, was used for transferring 10 μl of reaction mix and 5 μl of DNA (5 ng/μl) in a 96-well plate. In total, 25 ng (5 μl 5 ng/μl) of DNA sample was added to each reaction, and each sample was run in triplicate. All PCR reactions were performed on a SteponePlus Real-Time PCR System (Applied Biosystems, Milano, Italy). The average of the three T measurements was divided by the average of the three S measurements to calculate the average T:S ratio, i.e. the relative telomere length. A measure was considered acceptable if the standard deviation (SD) among triplicate measures was < 0.25. The coefficient of variation for the average T:S ratio of samples analyzed over three consecutive days was 8.5%, which was similar to the reproducibility originally reported for this method^4^.

### Sample size

Estimating that a significant correlation would be in the order of r = 0.80, we calculated that the sample to obtain statistical significance (α 0.01) should be n = 15 (power 0.9).

### Statistical analysis

Statistical analyses were performed with StastDirects software. Data are expressed as median, minimum and maximum values unless otherwise specified. Comparison between two groups was made using Mann–Whitney U test and (two-tailed) Paired t test for paired samples. Correlation was evaluated by simple linear regression models (Kendall’s rank correlation) in order to provide a measure of the strength of dependence between two variables. The effect of the leucocyte count (independent variable) on blood DNAmAge and TL measures (dependent variables) was also evaluated by simple linear regression models (Kendall’s rank correlation). The effect of gender (independent variable) on DNAmAge, AgeAcc and TL of RA and LA and of blood leucocytes (dependent variables) was also evaluated by simple linear regression models (Kendall’s rank correlation). Results were considered significant when a *p* value of < 0.05 was obtained.

## Supplementary information

Supplementary information

## Abbreviations

TL: Telomere length
LTL: Leucocytes telomere length
DNAmAge: DNA methylation age
AgeAcc: Age acceleration
RA: Right atrium
LA: Left atrium
DBD: Donors after brain death
OCS: Organ Care System
