# Supplementary material for: The biological age of the heart is consistently younger than chronological age
Source: Sci Rep. 2020 Jul 1;10:10752. doi: 10.1038/s41598-020-67622-1 (PMC7329913; doi:10.1038/s41598-020-67622-1)
Supplement: Supplementary file 1 — Supplementary information [file 41598_2020_67622_MOESM1_ESM.docx]

**The biological age of the heart is consistently younger than chronological age.**

Sofia Pavanello *^1,2^, Manuela Campisi^1^, Assunta Fabozzo ^2,3^, Giorgia Cibin ^3^, Vincenzo Tarzia ^3^, Giuseppe Toscano ^3^, Gino Gerosa ^2,3^.

^1^Occupational Medicine, Department of Cardiac, Thoracic, and Vascular Sciences and Public Health, University Hospital of Padova, Via Giustiniani, 2, 35128, Padova, Italy.

^2^Lifelab Program, Consorzio per la Ricerca Sanitaria-CORIS, Veneto Region, Via Giustiniani 2, 35128, Padova, Italy.

^3^Cardiac Surgery Unit, Department of Cardiac, Thoracic, and Vascular Sciences and Public Health, University Hospital of Padua, Via Giustiniani, 2, 35,128 Padova, Italy.

Authors have no disclosure

Word count: 3212

***Corresponding Author**

Sofia Pavanello

Unit of Occupational Medicine, Department of Cardiac, Thoracic, and Vascular Sciences and Public Health, University of Padova,

Via Giustiniani 2 - 35128 Padova, Italy

E-mail: sofia.pavanello@unipd.it

Tel.: +39 049 8216600; fax: +39 049 8212542

**Supplementary Material**

*DNA extraction from blood and tissue samples*

DNA extraction was performed on all samples of whole blood using an automated QIAcube System (Qiagen, Milano, Italy according to the DNAeasy Blood and Tissue kit (Qiagen, Milano, Italy procedure for high-throughput purification from human blood, following the manufacturer’s instructions and a customized protocol. In particular, 400 µL of whole blood from each sample were processed for DNA extraction. DNA extraction from cardiac tissue samples was also performed on QIAcube System (Qiagen, Milano, Italy) with some procedural differences compared to blood. In brief, a maximum of 25 mg of tissue sample was disrupted using TissueLyser II (Qiagen, Milano, Italy) system through high-speed shaking with beads, which beat and grind samples. The TissueLyser II system delivers thorough and rapid disruption of samples to fully release biomolecules, and also simultaneously homogenizes samples to facilitate subsequent purification procedures on QIAcube System (Qiagen, Milano, Italy) by using DNAeasy Blood and Tissue kit (Qiagen, Milano, Italy) following the manufacturer’s instructions. After extraction, DNA was quantified and checked for quality using QIAexpert, Quantification System (Qiagen, Milano, Italy).

*DNAmAge analysis*

DNAmAge was determined by analysis the methylation levels from selected markers using bisulfite conversion and Pyrosequencing methodology. This method is based on determination of methylation level of a set of five markers (ELOVL2 C1orf132, KLF14, TRIM59 and FHL2) in genomic DNA, as described [1] with some modifications relatively to the fact that the method was completely automated using the PyroMark Q48 Autoprep (Qiagen) as previous described [2]. Briefly, 2 μg DNA was submitted to bisulfite conversion: unmethylated cytosines in extracted DNA were converted to uracil using Epitect Fast DNA Bisulfite (Qiagen) following manufacturer’s instructions. An aliquot of template DNA was used for PCR amplification of selected markers using PCR primers including in AgePlexMono kit (Biovectis, Poland). Details of sites and sequences to analyze are reported in Table S1. PCR reactions were performed in 25 μL, comprising 0.2 μM of each primers, 20 ng of [template DNA](https://www.sciencedirect.com/topics/medicine-and-dentistry/dna-template), and PyroMark PCR Master Mix holding HotStarTaq [DNA Polymerase](https://www.sciencedirect.com/topics/medicine-and-dentistry/dna-polymerase), 1x PyroMark PCR Buffer and dNTPs. The amplification plan involved the preliminary [denaturation](https://www.sciencedirect.com/topics/biochemistry-genetics-and-molecular-biology/denaturation) step at 95°C for 10 min, followed by 40–45 cycles of denaturation (94°C for 30 s), annealing (54–56°C for 60 s) and extension (72°C for 90 s), and a final extension of 72°C for 10 min. Each PCR amplification contained negative PCR controls. 10 µl of PCR product were used for each pyrosequencing primer containing in AgePlex Mono kit (Biovectis) and loaded in a 48 well-plate (Pyromark Q48 Discs, Qiagen). Pyrosequencing was performed on Pyromark Q48 Autoprep instrument (Qiagen) using Pyromark Q48 Advanced Reagents (Qiagen) according to manufacturer’s instructions. The resulting Pyrograms generated by the instrument were automatically analyzed using Pyromark Q48 Autoprep Software (Qiagen). The level of methylation was expressed as percentage of methylated cytosines at the 5 CpG sites considered. The methylation percentages were inserted in an online calculator system accessible at [www.agecalculator.ies.krakow.pl](http://www.agecalculator.ies.krakow.pl), for estimation of biological age from DNA methylation analysis. The equation corresponds to a previously developed age prediction model [1]. All samples were analyzed 3 times for each marker, to verify the reproducibility of our results, and their average was utilized in the statistical testing. All samples were analyzed in two different days and coefficient of variation (CV) in replicate pyrosequencing runs was 0.5 %.

**Table S1. Details of** **markers, AgePlex sequences to analyze, CpG sites and location of the prediction model.**

| marker | Sequence to analyze | CpG site | Location (GRCh38) |
| --- | --- | --- | --- |
| ELOVL2 | CCRTAAACRTTAAACCRCCRCRCRAAACCRAC | C7 | Chr6: 11044661 |
| C1orf132 | AAATCTACRCAAACRACRATAAATAATCC | C1 | Chr1: 207823681 |
| TRIM59 | GGTTTGGYGYGGGAYGAGGYGAAGYGTYGG  TGGTYGAYGGTTTTTGAGGAATTATTTTTTATTT | C7 | Chr3: 160450199 |
| KLF14 | TYGYGTTTTTTTTTTTGTYGGYGAGTTAGGTA  ATGGTAATAGAG | C1 | Chr7: 130734355 |
| FHL2 | AGTTATYGGGAGYGTYGTTTTYGGYGTGGG  TTTTYGGGYGYGAGTTTYGGAYGAGGTTTGGG | C2 | Chr2: 105399288 |

**References**

1. Zbieć-Piekarska, R. et al. Development of a forensically useful age prediction method based on DNA methylation analysis. *Forensic Sci Int Genet.* **17**, 173-179 (2015).
2. Pavanello, S., Campisi, M., Tona, F., Dal Lin, C., Iliceto, S. Exploring Epigenetic Age in Response to Intensive Relaxing Training: A Pilot Study to Slow Down Biological Age. *Int J Environ Res Public Health.* **16**, 3074 (2019).
